# Secular temporal shifts in alexithymia among Chinese college students (2003–2024): a cross-temporal meta-analysis

**DOI:** 10.3389/fpsyg.2026.1945943

**Published:** 2026-09-25

**Authors:** Shizhen Yan, Feng Gao, Kexin Shi

**Affiliations:** 1 School of Health, Fujian Medical University, Fuzhou, China; 2 Mental Health Service Center, Yanshan University, Qinhuangdao, China; 3 School of Traditional Chinese Medicine, Beijing University of Chinese Medicine, Beijing, China

**Keywords:** alexithymia, college students, cross-temporal meta-analysis, psychological stress, social environment

## Abstract

Alexithymia constitutes a key indicator for assessing mental-health status among college students. Employing cross-temporal meta-analytic methods, the present study synthesizes repeated cross-sectional empirical data spanning 2003 to 2024 to map secular patterns of alexithymia in Chinese undergraduate samples and characterize observational temporal covariation between lagged aggregated macrosocial indicators and alexithymia-related traits. In total, 140 eligible studies involving 141723 undergraduate participants were incorporated; analyses were restricted strictly to datasets collected using the fully validated Chinese adaptation of the TAS-20 scale. Extensive between-study heterogeneity was observed for all alexithymia-related outcome measures. Univariate meta-regression detected significant positive temporal covariation of total alexithymia, Difficulty Identifying Feelings (DIF), and Difficulty Describing Feelings (DDF) with data-collection years, whereas Externally Oriented Thinking (EOT) exhibited no discernible temporal covariation. Multiple lagged macrosocial indicators showed bivariate observational covariation with elevated alexithymia scores. Multivariate adjusted meta-regression further revealed differentiated correlational profiles linking economic predictors to distinct alexithymia subdimensions. Supplementary sensitivity tests and publication-bias assessments were implemented to evaluate the robustness of these observational patterns, alongside explicit acknowledgment of methodological limitations intrinsic to observational meta-analytic research. Aggregated findings document secular increasing patterns in emotion-identification and emotion-description deficits across pooled college-student samples, as well as observational temporal covariation with multifaceted macrosocial transformations. The present results reflect population-level correlational covariation and provide no evidence for causal connections or intra-individual longitudinal shifts.

**Systematic review registration:**

This cross‑temporal meta‑analysis is observational social‑science evidence‑synthesis and falls outside the eligibility scope of the PROSPERO registry, which mainly accepts health‑intervention‑focused systematic reviews. The study protocol, full extracted dataset and reproducible analytical R‑scripts have been publicly archived on the Open Science Framework (OSF). Public accessible project URL: https://osf.io/4d5pt/.

## 1 Introduction

Alexithymia refers to a stable personality trait distinguished by core clinical manifestations: impaired capacity to recognize one’s own feelings, difficulty verbalizing emotional experiences, a tendency toward externally oriented cognitive processing, and inattention to internal affective states (Taylor, 2000). Sifneos (1996) even coined the phrase “the personality of our times” to highlight its pervasive prevalence amid modern social life. Accumulated empirical evidence has validated alexithymia as a robust transdiagnostic risk factor for poor psychological and behavioral adjustment. It exhibits stable positive correlations with chronic negative affective states including anxiety and depression, and elevates individual vulnerability to severe adverse outcomes such as substance misuse, deliberate self-harm, and interpersonal aggression (Liu and Wang, 2019; Morie et al., 2016; Zhang et al., 2020). Beyond clinical psychiatric risks, alexithymic traits also predict higher likelihood of academic or occupational burnout, rigid cognitive biases, and persistent post-traumatic stress symptoms (Edwards and Lowe, 2021; Hemming et al., 2021; Ledermann et al., 2020). Though alexithymia is categorized as a dimensional personality disposition rather than a definite mental disorder, pronounced alexithymic deficits can severely hinder long-term socioemotional and cognitive development (Lumley et al., 2007). Moreover, the three-year COVID-19 public health crisis, characterized by prolonged isolation and chronic existential stress, is hypothesized to aggravate population-level alexithymic symptoms, sparking growing research interest across clinical and social psychology domains (Wang et al., 2021).

### 1.1 Theoretical background and research gap

China has undergone far-reaching socioeconomic restructuring over the past two decades, accompanied by drastic shifts in economic operation modes, cultural value orientations, and daily lifestyles. Large-scale macro-social changes generate heterogeneous psychological impacts across different youth cohorts (Cai et al., 2020). As the core reserve of future social construction, college students are situated in a critical developmental transition period, simultaneously confronted with overlapping stressors from academic competition, uncertain career prospects, and interpersonal adaptation (Xin et al., 2011). Prior cross-temporal meta-analyses have documented systematic secular changes in Chinese undergraduates’ psychological profiles, covering trait anxiety, achievement motivation and time-management tendencies (Chen and Sun, 2022; Xin et al., 2011). Nevertheless, extant research on youth alexithymia is overwhelmingly dominated by discrete cross-sectional surveys, and few quantitative syntheses have tracked long-term shifts in alexithymia among college students across two decades, creating a clear empirical gap addressable through cross-temporal meta-analysis.

Bronfenbrenner’s social-ecological system theory and the stress-process model provide interpretive grounding for the present investigation. The social-ecological framework posits that individual emotional cognition is shaped by multi-layered environments, where regional economic, cultural, and public-health macro-systems exert long-term influences on emotional socialization (Bronfenbrenner, 1979). Complementing this framework, stress-process theory suggests chronic structural pressure and large-scale public-health emergencies can disrupt routine emotion-regulation functioning and gradually erode capacities for identifying and describing inner feelings (Pearlin et al., 1981). Decades of theoretical and alexithymia-focused empirical work further underscore its social-developmental origins, noting that sociocultural context, economic standing, and early-family atmosphere constitute key formative predictors of alexithymic traits (Zhang et al., 2011).

A recent updated meta-analysis on alexithymia has mapped gender-related differences across international samples and summarized cross-sectional effect-size heterogeneity (Mendia et al., 2024). Even so, this and other existing syntheses focus primarily on static cross-sectional pooled estimates and demographic subgroup contrasts. To our knowledge, few meta-analytic efforts have systematically quantified secular cohort-level temporal shifts in alexithymia across two decades within a single cultural context, nor examined observational temporal correlations between societal-level macrosocial indicators and alexithymia-related traits. Drawing on Bronfenbrenner’s social-ecological system theory and the stress-process model, these theoretical perspectives motivate our observational correlational hypotheses: multiple layers of societal-level contextual features may correspond to shifts in alexithymia-related traits across youth cohorts. We emphasize that these are tentative observational hypotheses, not causal predictions, given the inherent limitations of aggregated meta-analytic data. This important empirical gap motivates the present cross-temporal meta-analysis.

### 1.2 Limitations of existing evidence

Despite substantial research examining alexithymia among Chinese college students, the existing empirical evidence remains fragmented and inconsistent. Cross-sectional studies have reported divergent prevalence estimates, with some documenting mild-to-moderate alexithymic traits in undergraduate samples (Fang et al., 2020; Hou et al., 2021), while others identify clinically concerning elevated symptom levels (Jiang et al., 2023; Zhou et al., 2022). Gender differences across the three core alexithymia dimensions—Difficulty Identifying Feelings (DIF), Difficulty Describing Feelings (DDF), and Externally Oriented Thinking (EOT)—also vary inconsistently across individual investigations, whereas recent meta-analyses focusing on Chinese youth populations suggest no stable, statistically significant gender disparities (Li et al., 2024; Wang et al., 2024; Zhong et al., 2026).

These empirical inconsistencies arise from pervasive methodological limitations in primary studies. Variations in university and major sampling coverage, inconsistent measurement tools across research teams, and widespread reliance on static cross-sectional designs collectively restrict reliable population-level inference. Conventional one-off sampling cannot address temporal bias and fails to capture long-term developmental trends in youth emotional traits. Collectively, these disjointed findings tentatively imply a possible rise in alexithymia levels among Chinese college students over recent decades, which the present cross-temporal meta-analysis aims to systematically verify.

### 1.3 Aims and contributions

This study conducted a cross delimiter temporal meta-analysis on alexithymia among Chinese college students to resolve the methodological limitations and empirical gaps identified in existing research. The literature search included all eligible studies published up to the end of 2025. Five sets of macrosocial indicators were adopted to capture economic status, family structure and social environment, with each index assumed to correlate with temporal changes in college students’ emotional functioning. This study recognizes potential confounding factors that cannot be completely excluded from the current dataset. Variations in scale application, sample composition, university types and regional participant distribution across different time periods may interfere with the interpretation of temporal trends.

This analytical approach clearly distinguishes multiple conceptual definitions in temporal research. Cross delimiter temporal secular trends reflect aggregated cohort level patterns based on cross sectional data. Cohort effects describe generational differences among college students’ groups from different periods. Individual longitudinal change captures intra personal variation through continuous tracking of the same participants. The present work focuses exclusively on aggregated cohort level temporal patterns and does not involve individual level dynamic changes.

This study systematically explored long term developmental trends in overall alexithymia and its three subdimensions of difficulty identifying feelings, difficulty describing feelings and externally oriented thinking among successive college student cohorts. It further analyzed temporal correlations between multi-year lagged macrosocial indicators and alexithymia traits, and explored potential changes in college students’ emotional characteristics associated with the COVID 19 pandemic through subgroup analysis. This quantitative synthesis can effectively compensate for sampling bias prevalent in independent small scale studies by generating reliable population level observational results. The findings also provide empirical evidence for optimizing college mental health services and psychological education strategies.

## 2 Methods

### 2.1 Literature search

#### 2.1.1 Inclusion and exclusion criteria

Drawing on established protocols of prior cross-temporal meta-analyses of Chinese college student psychology (Xin et al., 2011; Xin and Wang, 2019; Chen and Sun, 2022), we formulated uniform inclusion criteria consistent with the present research aims:

- Adopted the full unmodified Chinese version of the 20-item Toronto Alexithymia Scale (TAS-20) without item deletion or scale revision. This strict inclusion criterion reduces measurement heterogeneity introduced by modified or non-validated scale adaptations, but it may also narrow the evidence base and introduce potential selection bias;
- Reported complete quantitative outcome data, including sample size and TAS-20 total/subdimension means;
- Targeted undergraduate students studying at mainland Chinese higher education institutions;
- Published online or in print by December 2025;
- In cases of overlapping samples across multiple works by the same research team, only the study with the most comprehensive raw data was retained.

Exclusion criteria were as follows: (a) Studies employing non-TAS-20 alexithymia measurement tools; (b) Papers lacking accessible mean score or sample statistics; (c) Samples consisting primarily of non-mainland Chinese populations, postgraduates, or clinical patient groups; (d) Duplicate datasets published across different journals.

#### 2.1.2 Database search and screening process

Systematic literature retrieval was conducted across six mainstream academic databases, including CNKI, Wanfang Data, VIP Chinese Journal Database, CSSCI, Web of Science, and Google Scholar. The final search was completed on December 31, 2025, covering publications spanning from January 2003 to December 2025. The starting time point was determined based on the official validation and publication of the Chinese version of the TAS-20 scale (Yi et al., 2003; Yuan et al., 2003). Consistent keyword combinations containing free-text terms and wildcard operators were applied separately for Chinese and English databases. Detailed search strings for each database are provided in Supplementary Table S1 to ensure full methodological reproducibility.

Search term formulation followed standardized retrieval frameworks adopted in prior cross-temporal meta-analyses on Chinese college students’ psychological traits. The current study did not implement forward or backward citation tracking, and gray literature beyond formal academic publications was not included in the screening procedure. Eligible literature encompassed formally published peer-reviewed journal articles and official dissertations. This retrieval strategy may introduce potential publication bias, as unpublished and non-peer-reviewed studies with non-significant or small effect sizes were not incorporated into the analytical dataset.

The initial electronic search generated 749 independent literature records. Sequential title and abstract screening, followed by rigorous full-text evaluation, led to the exclusion of 609 ineligible studies based on pre-defined screening criteria. The final analytical dataset comprised 140 qualified studies, with a cumulative sample of 141,723 undergraduate participants. Four included studies recruited a small number of postgraduate participants alongside undergraduate samples, yet these studies were still retained in the final analysis because college students constituted over 80 percent of their total research samples. All eligible studies were systematically coded and summarized in Supplementary Table S2 to support data transparency and reproducibility.

Each included study was coded with its corresponding data collection year to support subsequent temporal analyses. For studies without explicit survey time information, collection year was estimated by subtracting 2 years from the publication year, following the classic coding convention widely adopted in cross-temporal meta-analyses (Twenge, 2000). The coded data collection years ranged from 2003 to 2024 across the full dataset. The entire literature screening procedure strictly complied with the PRISMA 2020 reporting guidelines (Page et al., 2021), and the complete screening workflow is illustrated in the PRISMA flow diagram presented in Figure 1.

**Figure 1:**
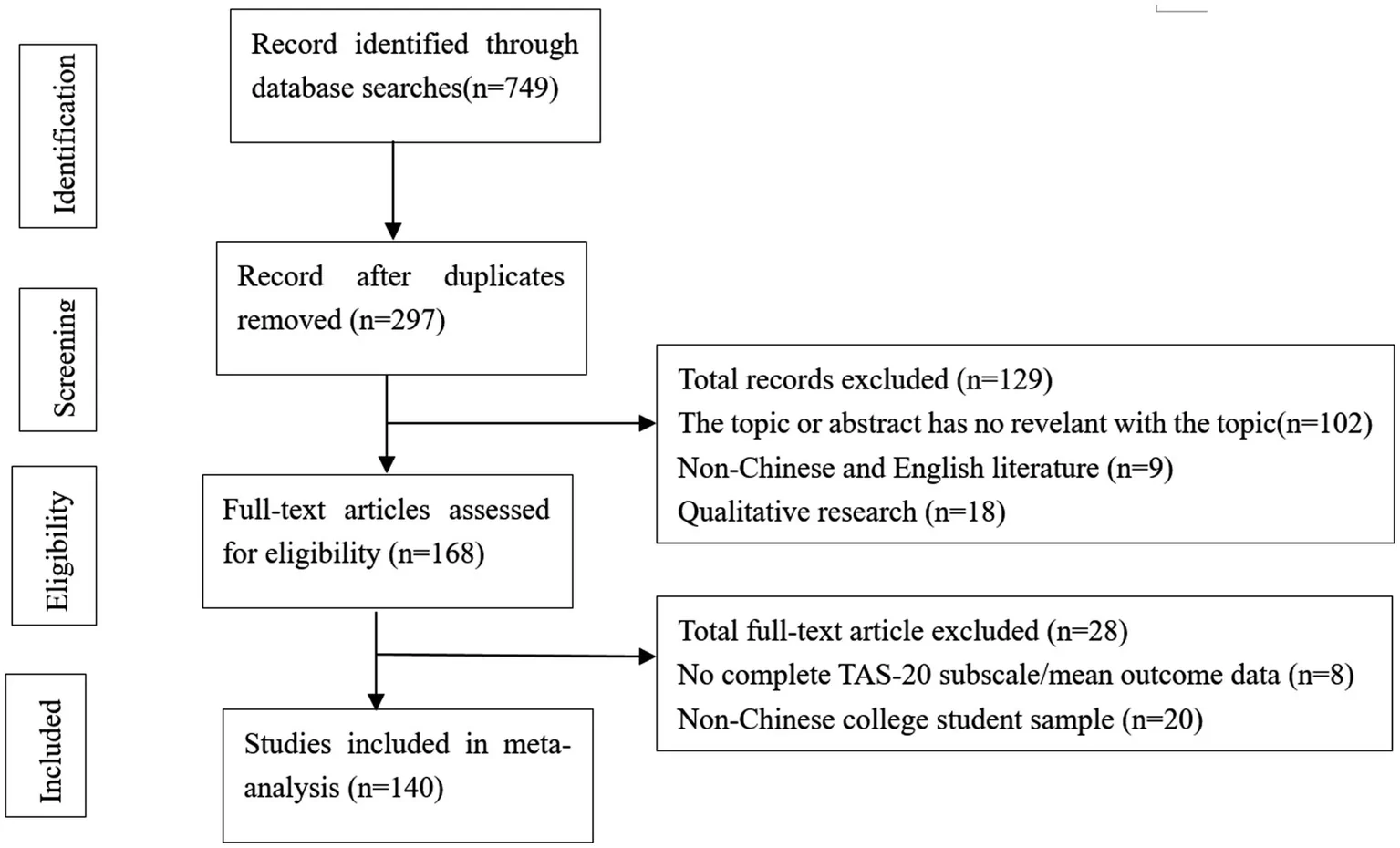
PRISMA diagram of the search strategies and inclusion process.

### 2.2 Alexithymia measurement tool (TAS-20)

The TAS-20 contains 20 items rated on a 5-point Likert scale ranging from 1 (“completely disagree”) to 5 (“completely agree”). The scale comprises three subscales: Difficulty Identifying Feelings (7 items), Difficulty Describing Feelings (5 items), and Externally Oriented Thinking (8 items). The Chinese translation of TAS-20 has been localized and validated among Chinese youth populations, with good reliability and validity reported in existing empirical studies (Yi et al., 2003; Yuan et al., 2003).

### 2.3 Selection of macro-social indicators

Based on established empirical frameworks from prior cross-temporal meta-analyses (Xin et al., 2011; Xin and Wang, 2019; Chen and Sun, 2022) and the theoretical foundations of the current study, five macro-social indicators across three core domains were selected as lagged predictive variables. The included indicators cover key societal dimensions closely linked to college students’ emotional development, including economic conditions, family and interpersonal contexts, and broader social operational environments. The detailed indicator classification is presented as follows:

- Economic conditions: per capita GDP and average housing price;
- Interpersonal and family context: crude divorce rate;
- Social operational environment: unemployment rate and internet penetration rate.

All indicator data were retrieved from official annual China Statistical Yearbook datasets. Given the substantial numerical differences in raw indicator units, linear rescaling was conducted prior to meta-regression analysis to standardize coefficient interpretation. Regional per capita GDP measured in 100 million RMB was divided by 10,000 to reflect increments per trillion RMB, while average housing price measured in RMB was divided by 1,000 to represent changes per 1,000 RMB increase in housing costs. This linear transformation only adjusted the numerical magnitude of regression coefficients and standard errors, without altering statistical significance, *Q_M_* test outcomes, or heterogeneity explanation magnitude. To explore temporal associations between societal changes and alexithymia traits, separate meta-regression models with three-year and five-year lag windows were constructed for all macro-social indicators.

### 2.4 Methodological quality assessment of included studies

The Checklist for the Methodological Quality Assessment of Studies (CMQAS; Prins et al., 2002) was adopted to evaluate the methodological quality of included studies. This checklist comprises 15 binary-scored criteria covering three dimensions: external validity (6 items), internal validity (6 items), and informativity (3 items). While the original publication suggested that the three informativity-focused items should not contribute to methodological-quality total scores, following common practice in Chinese meta-analyses of college-student mental health (Shi et al., 2023), all 15 items were scored in the present study. Each item received a score of 1 when the criterion was satisfied and 0 when it was not satisfied; higher total scores indicate superior overall study quality.

### 2.5 Literature coding and data aggregation

Literature screening, data extraction, and methodological quality assessment were independently completed by two authors. Inter-rater consistency was quantitatively evaluated using Cohen’s Kappa coefficient for categorical eligibility judgments and the intra-class correlation coefficient (ICC) for continuous coded variables, including sample size, mean scores, standard deviations, and overall CMQAS quality scores. Any coding discrepancies were initially resolved through mutual discussion, and remaining inconsistencies were finally adjudicated by the corresponding author. Reliability analyses indicated excellent inter-rater agreement: Cohen’s Kappa reached 0.86 for study screening, and the ICC value was 0.92 (95% CI [0.87, 0.95]) for quantitative data extraction and methodological quality evaluation. All cleaned study characteristics and extracted analytical data are fully documented in Supplementary Table S2, covering all variables adopted for subsequent meta-analytic analyses.

Referring to standardized coding protocols established in previous cross-temporal meta-analyses (Xin et al., 2011; Chen and Sun, 2022), all 140 included studies were systematically coded with unified criteria. Detailed coding procedures are described as follows:

- Each eligible study was assigned an exclusive identification number. Basic analytical information, including sample size, TAS-20 total and subdimension means, standard deviations, publication year, and data collection year, was uniformly imported into the analytical database.
- Several included studies only reported segmented subgroup statistics rather than overall sample outcomes. Common subgroup divisions included gender differences and romantic experience status. To acquire unified overall sample indicators for meta-analytic synthesis, aggregated mean and standard deviation values were calculated using conventional meta-analytic pooling formulas (Equations 1, 2). This aggregation procedure assumes moderate correlation across within-study subgroups. Given the absence of original subgroup correlation coefficients in primary publications, this core assumption could not be empirically verified, which constitutes an inherent limitation of the current data aggregation process.
- Additional contextual variables were also extracted and coded for further analysis, including publication type, female sample proportion, and student major category.

$$\bar{x}=\sum x_{i}n_{i}/\sum n_{i}$$ (1)

$$S_{T}=\sqrt{[\sum n_{i}S_{i}^{2}+\sum n_{i}{\left(x_{i}-{\bar{x}}_{i}\right)}^{2}]/\sum n_{i}}$$ (2)

### 2.6 Statistical analyses

All statistical analyses were conducted using R 4.3.2 with the metafor package. The core analytical procedures were implemented as follows:

- Random-effects meta-analyses with restricted maximum likelihood (REML) estimation were performed to calculate pooled weighted mean scores for the overall TAS-20 scale and its three subdimensions. Heterogeneity across studies was evaluated using the *I^2^* statistic and Cochran’s *Q* test. *Z*-tests were further applied to compare pooled mean values against scale clinical cutoffs and theoretical midpoints.
- Descriptive statistics were generated for CMQAS methodological quality scores and average sample sizes. Primary-study sampling variance for each effect size was calculated using the formula *v_i_* = *SD*^2^/*N*, which quantifies sampling variation for raw study means and supports weighted meta-analytic modeling. While REML-based random-effects meta-regression further estimates the between-study variance component *τ^2^* to account for cross-study heterogeneity, the current analytical framework does not explicitly model potential non-independence of multiple effect sizes sourced from a single study, which may slightly underestimate standard errors. Robust variance estimation was not adopted in the present study, as nearly all included studies contributed only one effect size; such sparse cluster structures compromise the statistical performance of robust variance estimation. This methodological limitation is elaborated in the final discussion section.
- Univariate weighted meta-regression models were fitted to examine long-term temporal trends in college students’ alexithymia across data collection years.
- Subgroup meta-regression analyses were stratified by three distinct pandemic stages including pre-pandemic, pandemic-era, and post-pandemic periods, to identify differentiated temporal patterns across public health phases.
- Hierarchical meta-regression models were constructed to disentangle confounding effects and examine macrosocial correlates of alexithymia traits. Given severe multicollinearity among the five selected macrosocial indicators, simultaneous inclusion of all predictors within a single multivariate framework was statistically infeasible. Accordingly, all macrosocial indicators were first examined using independent univariate meta-regressions under both three-year and five-year lag conditions to explore crude bivariate temporal associations with alexithymia dimensions. A parsimonious multivariate adjusted model was further established to control for core confounding variables, incorporating data collection year, female sample proportion, three-year lagged per capita GDP, and three-year lagged unemployment rate. This limited predictor set was selected to balance theoretical rationality and statistical stability.

Female proportion was adjusted following classic cross-temporal meta-analysis protocols (Twenge, 2000; Xin and Wang, 2019), as shifting gender ratios across sampling years may generate spurious temporal trends without statistical control. Consistent with recent meta-analyses on Chinese college student populations (Wang et al., 2024; Zhong et al., 2026), stable gender disparities in alexithymia were not observed, and weak correlations between female ratio and economic indicators avoid multicollinearity interference in the adjusted model.

The three-year lag window adopted for economic variables conforms to mainstream analytical conventions in domestic cross-temporal meta-analyses (Xin et al., 2011; Chen and Sun, 2022; Zhang et al., 2020). This time frame aligns with the undergraduate academic cycle and captures recent economic stress exposure for college students, while five-year lagged indicators reflect long-term cumulative societal impacts. Concurrent inclusion of dual lagged terms for identical economic metrics would induce severe multicollinearity and biased coefficient estimation, consistent with core assumptions of the stress process model (Pearlin et al., 1981) and Twenge’s cross-temporal analytical framework (Twenge, 2000). Housing price, divorce rate, and internet penetration rate were excluded from the multivariate model due to strong collinearity with per capita GDP, which would undermine coefficient robustness and model stability.

- Systematic robustness checks were implemented to verify the reliability of temporal and macrosocial correlational findings. Egger’s regression test was conducted to detect potential publication bias, and leave-one-out sensitivity analysis was performed to examine the stability of overall results. An additional sensitivity analysis excluding studies with partial postgraduate participants was conducted to validate result robustness, with corresponding outcomes reported in Supplementary Table S3.

### 2.7 Data availability

The full study-level dataset supporting this meta-analysis is available in Supplementary Table S2. R analytical scripts for temporal modeling, multivariate meta-regression, sensitivity analyses, and publication-bias diagnostics have been deposited on the Open Science Framework (OSF). The OSF repository also hosts an archival backup copy of the extracted dataset (Data Extraction Table of Included Studies.xlsx). For peer-review assessment and independent replication, a private view-only OSF link is provided: https://osf.io/4d5pt/overview?view_only=f9b439fe784d43949ff2368d5cdbd070. This archived project resource includes complete descriptive information for all included studies, standardized meta-analytic input data, and reproducible code for all temporal, multivariate, sensitivity, and publication-bias analyses.

## 3 Results

### 3.1 Results of literature quality evaluation

The CMQAS checklist was adopted to assess the methodological rigor of all 140 included studies. Complete valid quality ratings were obtained for every paper with no missing CMQAS scores. The average methodological quality score was 12.0 ± 1.3, with individual scores ranging from 8.0 to 14.0, which indicated moderate to high overall methodological quality across the full sample.

Among all 140 studies, 132 reported complete total TAS-20 mean and variance data for total alexithymia pooled analysis, while the remaining 8 articles lacked total score statistics but provided valid separate effect sizes for the three subdimensions (DIF, DDF, EOT). All 140 datasets were retained for subdimension-specific meta-regression and trend analysis.

### 3.2 Pooled alexithymia means versus scale cutoffs and midpoints

Random-effects meta-analysis with REML estimation was performed to synthesize weighted mean scores for the TAS-20 total scale and its three subdimensions. Marked between-study heterogeneity was observed across all four outcomes (all *I^2^* > 99%, all *p* < 0.001), supporting the use of random-effects models. Pooled estimates, standard errors, 95% confidence intervals, and heterogeneity statistics are summarized in Table 1. 95% prediction intervals are additionally reported in Supplementary Table S4 to illustrate the expected distribution of true effect sizes across individual primary studies. Custom z-tests were implemented to compare pooled means against established clinical cut-off values and theoretical scale midpoints.

**Table 1:** Pooled means, heterogeneity indices for TAS-20 total and three subdimensions.

Outcome	*k*	M	*SE*	τ^2^	*I^2^*(%)	*Q* Statistic	*p* (*Q* test)
TAS-20	132	54.29	0.37	17.86	99.58	68100.71	<0.001
DIF	77	18.90	0.24	4.44	99.79	55074.58	<0.001
DDF	77	14.99	0.31	7.39	99.88	122813.08	<0.001
EOT	77	21.73	0.31	7.50	99.93	126404.79	<0.001

k, number of eligible studies with complete data for this outcome; SE, standard error; τ^2^, between-study variance; I^2^(%), proportion of total variance attributable to heterogeneity; Q, Cochran’s heterogeneity statistic; p (Q test), *p*-value of Cochran’s Q test for heterogeneity.

The pooled total TAS-20 mean was significantly higher than the clinical cut-off of 51 (*Z* = 8.89, *p* < 0.001), indicating mild overall alexithymic tendencies among contemporary college students. For the DIF subscale, the pooled mean was significantly lower than the theoretical midpoint of 21 (*Z* = −8.71, *p* < 0.001), reflecting relatively strong ability to identify internal emotional states. No statistically meaningful deviation from the neutral midpoint of 15 emerged for the DDF dimension (*Z* = −0.02, *p* = 0.987), which points to average capacity for verbalizing inner feelings. For the EOT subdimension, the pooled mean was substantially below its theoretical midpoint of 24 (*Z* = −7.25, *p* < 0.001), demonstrating a weak tendency toward externally oriented cognitive styles.

Given the extremely high heterogeneity across included studies, the single pooled absolute mean should be interpreted with caution and cannot be treated as a generalized population estimate. For this reason, primary conclusions of the present study emphasize temporal meta-regression slopes that capture cohort-level secular trends, rather than the magnitude of pooled absolute alexithymia scores. Several potential sources of heterogeneity, such as university sampling frameworks, regional socioeconomic variation, and subtle procedural differences in scale administration, could not be systematically coded from primary study reports.

### 3.3 Univariate meta-regression: secular trends across data collection years

Following the overall pooled analysis, mixed-effects meta-regression was conducted with data collection year specified as the sole moderator to examine long-term temporal changes in alexithymia. Substantial residual heterogeneity remained across all four fitted models (all *I^2^* > 99%, all *ps* < 0.001). Complete regression coefficients, confidence intervals, and moderator test statistics are summarized in Table 2. The total TAS-20 score demonstrated a significant positive temporal trend: each incremental data-collection year corresponded to a mild rise in alexithymia, and data-collection year accounted for 7.80% of the between-study variance. Comparable significant ascending trends emerged for the DIF and DDF subscales, with temporal change explaining approximately one-fifth of cross-study heterogeneity for both dimensions. In sharp contrast, no statistically reliable linear temporal shift was detected for the EOT subscale; data-collection year explained only 1.11% of residual heterogeneity, and the corresponding regression coefficient failed to reach statistical significance.

**Table 2:** Univariate meta-regression: secular trends.

Outcome	*k*	*β* (year)	*SE*	95% *CI*	*Q_M_*	*p (Q_M_)*	*R^2^* (%)
TAS-20	132	0.28	0.08	[0.12, 0.44]	12.12	0.0005	7.80
DIF	77	0.28	0.05	[0.17, 0.38]	26.68	<0.001	25.46
DDF	77	0.33	0.07	[0.19, 0.46]	21.09	<0.001	21.03
EOT	77	0.11	0.08	[−0.05, 0.26]	1.85	0.1741	1.11

β (year), unstandardized regression coefficient for data collection year; SE, standard error of coefficient; CI, 95% confidence interval for β; Q_M_, omnibus test statistic for moderator effect; p (Q_M_), *p*-value of moderator significance test; R^2^ (%), percentage of residual between-study heterogeneity explained by collection year. Residual between-study heterogeneity remained substantial for all outcomes even after accounting for data-collection year. Full pooled effect-size statistics and 95% prediction intervals are reported in Supplementary Table S4.

Cohort-level secular temporal patterns for the total TAS-20 score and its three subdimensions spanning 2003–2024 are illustrated in Figures 2, 3, providing visual confirmation of the numerical findings reported in Table 2. Distinct upward linear trends are observable for total alexithymia, DIF, and DDF, represented by positive fitted blue slopes alongside narrow 95% gray confidence bands that lie entirely above zero. For EOT, by contrast, the near-horizontal fitted line coupled with a wide confidence interval visually supports the absence of a meaningful secular trend within the observation window. The variable size of individual scatter points reflects the distribution of primary-study sample sizes across calendar years, showing that large-sample investigations were evenly distributed over the 2003–2024 study period.

**Figure 2:**
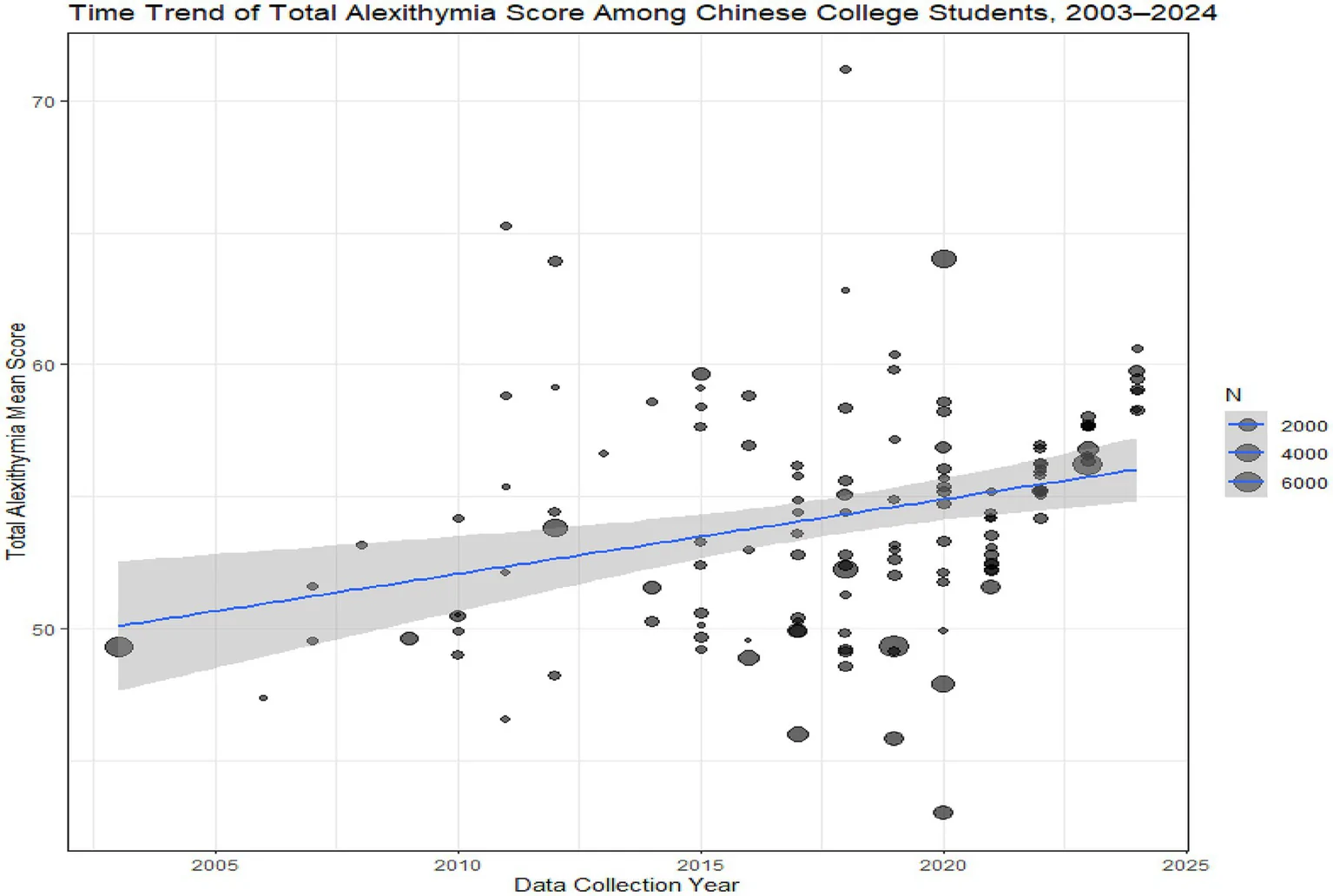
Longitudinal time trend of total alexithymia mean score among Chinese college students, 2003–2024. Blue solid line indicates the fitted temporal meta-regression slope; gray shaded band represents the 95% confidence interval of the trend estimate. Dot size is proportional to each study’s sample size.

**Figure 3:**
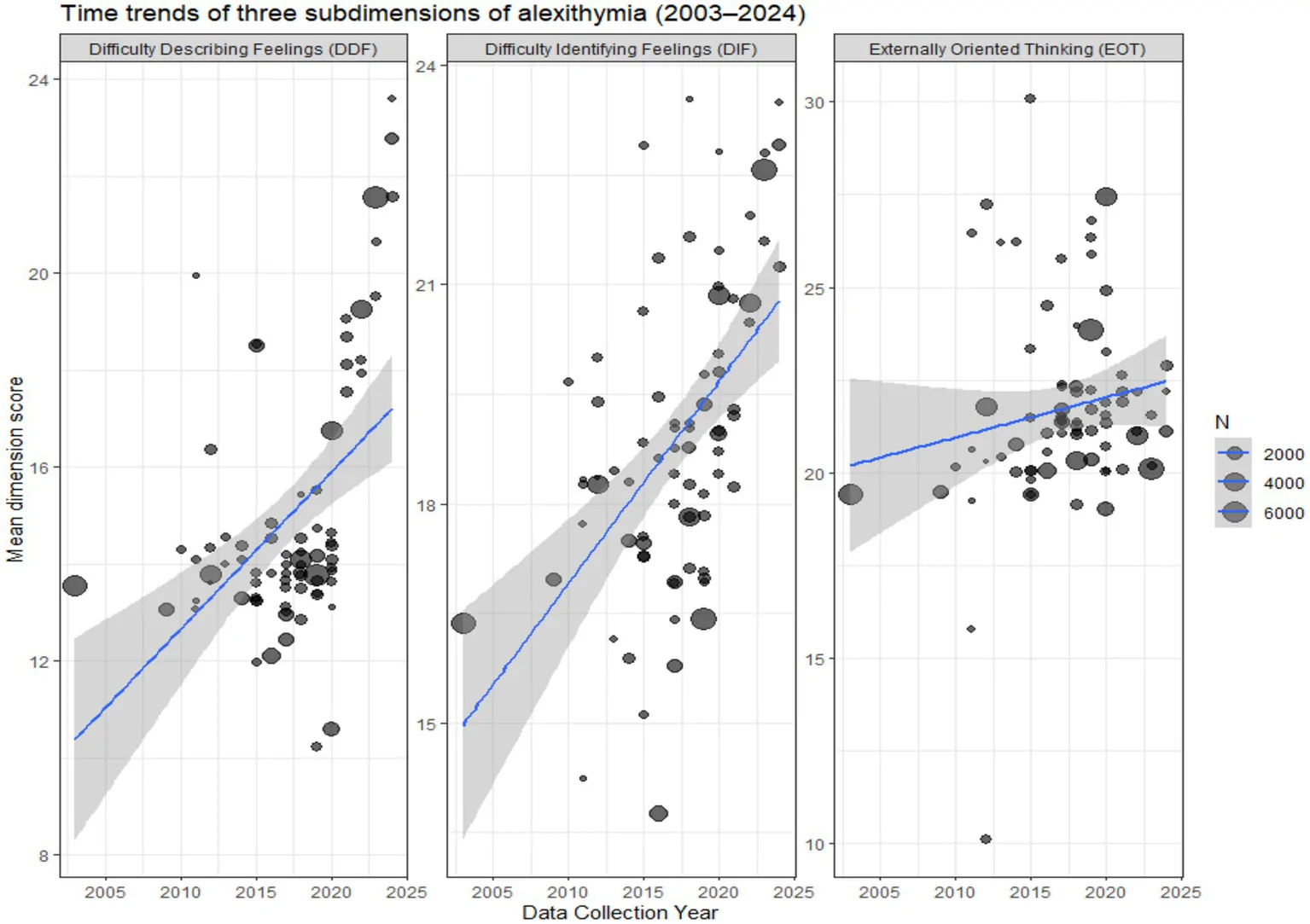
Time trends of three subdimensions of alexithymia among Chinese college students, 2003–2024. Blue solid lines represent the meta-regression linear trend; gray shaded areas denote 95% confidence intervals of the fitted slope. The size of each dot corresponds to the sample size of each individual study.

Prediction intervals were computed for all pooled random-effects models to describe the expected distribution of true effect sizes across future independent investigations. While the overall pooled means yielded relatively narrow confidence intervals, 95% prediction intervals were markedly wide for every analyzed outcome. This pattern aligns with the extremely high between-study heterogeneity observed across models (*I^2^* > 99.5%).

Although data-collection year explained a moderate-to-large share of between-study variance, with *R^2^* values ranging from 4.21 to 50.57% across alexithymia dimensions, considerable residual heterogeneity remained unexplained. Additional potential sources of heterogeneity involve divergent Chinese-language adaptations of the TAS-20 scale, variations in university-level sampling strategies, regional socioeconomic disparities, and participant-level demographic attributes. Many of these contextual variables could not be reliably extracted from published primary-study reports.

Given such extreme heterogeneity, interpretation of absolute pooled mean scores requires caution, and direct comparisons with theoretical scale cut-offs carry limited substantive interpretive utility. The present study prioritizes reporting secular temporal trends over absolute alexithymia magnitude for the main analytical output. Complete pooled statistics incorporating prediction intervals are provided in Supplementary Table S4.

### 3.4 Univariate meta-regression for separate macrosocial indicators

These meta-regression analyses of macrosocial indicators serve purely exploratory observational purposes. Aggregated macrosocial indicators introduce risks of ecological-inference fallacy. Strong inter-correlations among time-varying societal variables and temporal confounding cannot be fully ruled out within the present analytical framework. Lagged model specifications do not verify temporally ordered causal effects. All reported associations are interpreted as correlational in nature. Given the considerable number of tested predictors, two distinct lag windows, and four alexithymia-related outcome measures, these analyses face elevated risks of inflated family-wise Type-I error. Formal correction for multiple comparisons was not performed. For this reason, all outputs from macrosocial meta-regressions are regarded as exploratory observational patterns instead of confirmatory hypothesis-testing findings.

Building on the unadjusted temporal meta-regression using survey collection year alone, a set of independent univariate mixed-effects meta-regression models was fitted to evaluate bivariate zero-order associations between alexithymia subdimensions and five macrosocial predictors (lagged GDP, unemployment rate, divorce rate, housing price, internet penetration). Models were run separately for the 3-year and 5-year lag windows. Each predictor was included as the sole moderator within its respective model. Linear scaling transformations were applied to macroeconomic variables with large raw numerical values prior to regression to facilitate coefficient interpretation. Lagged housing price (raw unit: RMB) was divided by 1000, so that resulting coefficients correspond to alexithymia score shifts per 1000-yuan increase in housing price. Regional GDP (raw unit: 100 million RMB) was divided by 10000 to express values in trillion-RMB units; coefficients thus reflect score variation linked to each one-trillion-yuan rise in regional total output. Such linear rescaling alters only coefficient magnitude and standard errors, while leaving significance tests, *Q_M_* statistics, and the explained-heterogeneity *R^2^* values unaffected.

Table 3 presents statistically significant macrosocial predictors for total TAS-20, DIF, and DDF. Lagged GDP displayed consistent positive zero-order associations with total TAS-20, DIF, and DDF under both lag windows, whereas no corresponding association emerged for EOT. For the three alexithymia dimensions showing positive associations, lagged unemployment rate, housing price, and internet penetration all yielded positive correlational signals. The lagged divorce rate showed positive associations with total TAS-20 and DDF only under the 5-year lag specification, while both 3-year and 5-year lagged divorce rates reached statistical significance for DIF. Among all examined predictors, the 3-year lagged unemployment rate accounted for the largest proportion of residual heterogeneity for DDF (*R^2^* = 61.92%), constituting the strongest observed bivariate correlate for difficulty describing feelings. Several predictors returned non-significant bivariate associations and are not listed in Table 3: the 3-year lagged divorce rate for total TAS-20 (*p* = 0.346), the 3-year lagged divorce rate for DDF (*p* = 0.629), and all macrosocial predictors for EOT (all *ps* > 0.05). Taken together, none of the examined socioeconomic and social-context variables displayed detectable associations with externally oriented thinking. This observational pattern suggests that societal-level socioeconomic factors may correlate specifically with difficulties in identifying and describing emotions, rather than the tendency to avoid internal emotional experience. Across all statistically significant predictors, results derived from 3-year and 5-year lag models exhibited high consistency in coefficient direction and statistical significance, demonstrating stability of these observed bivariate patterns across alternative lag-time specifications.

**Table 3:** Univariate meta-regression: alexithymia and macrosocial correlates.

Outcome	*k*	Predictor	*β*	*SE*	95% *CI*	*p*	*Q_M_*	*R^2^* (%)
TAS-20	128	GDP_Lag3	0.07	0.02	[0.03, 0.11]	<0.001	11.49	7.4
		GDP_Lag5	0.07	0.02	[0.03, 0.11]	<0.001	11.19	7.19
		Unemp_Lag3	2.45	0.71	[1.06, 3.85]	<0.001	11.87	7.73
		Unemp_Lag5	3.98	1.02	[1.98, 5.98]	<0.001	15.2	9.92
		Divorce_Lag5	1.85	0.58	[0.7, 2.99]	0.002	10	6.39
		HousePrice_Lag3	0.6	0.2	[0.3, 0.9]	<0.001	12.59	8.12
		HousePrice_Lag5	0.7	0.2	[0.3, 1.1]	<0.001	13.57	8.75
		Internet_Lag3	0.08	0.02	[0.03, 0.12]	<0.001	12.8	8.24
		Internet_Lag5	0.07	0.02	[0.02, 0.11]	0.003	9.02	5.72
DIF	77	GDP_Lag3	0.06	0.01	[0.04, 0.08]	<0.001	27.32	25.93
		GDP_Lag5	0.06	0.01	[0.04, 0.08]	<0.001	27.83	26.33
		Unemp_Lag3	2.36	0.52	[1.35, 3.38]	<0.001	20.8	20.97
		Unemp_Lag5	2.96	0.75	[1.49, 4.43]	<0.001	15.62	16.31
		Divorce_Lag3	1.05	0.44	[0.18, 1.92]	0.018	5.58	5.71
		Divorce_Lag5	1.93	0.38	[1.19, 2.68]	<0.001	26.17	25.09
		HousePrice_Lag3	0.6	0.1	[0.4, 0.8]	<0.001	32.02	29.25
		HousePrice_Lag5	0.7	0.1	[0.5, 0.9]	<0.001	32.54	29.6
		Internet_Lag3	0.07	0.02	[0.04, 0.1]	<0.001	24.28	23.64
		Internet_Lag5	0.06	0.01	[0.04, 0.09]	<0.001	19.52	19.74
DDF	77	GDP_Lag3	0.08	0.02	[0.04, 0.12]	<0.001	27.21	25.79
		GDP_Lag5	0.08	0.01	[0.05, 0.11]	<0.001	32.96	29.76
		Unemp_Lag3	5.15	0.46	[4.24, 6.06]	<0.001	123.5	61.92
		Unemp_Lag5	5.36	0.86	[3.67, 7.06]	<0.001	38.7	33.25
		Divorce_Lag5	2.34	0.5	[1.37, 3.31]	<0.001	22.4	21.97
		HousePrice_Lag3	0.8	0.1	[0.5, 1.1]	<0.001	33.18	29.92
		HousePrice_Lag5	0.9	0.2	[0.6, 1.2]	<0.001	35.69	31.51
		Internet_Lag3	0.09	0.02	[0.05, 0.13]	<0.001	21.37	21.26
		Internet_Lag5	0.07	0.02	[0.03, 0.11]	<0.001	13.39	14.11

Lagged GDP is scaled per trillion RMB (raw unit: 100 million RMB); lagged housing price is scaled per 1000 RMB. Only predictors with *p* < 0.05 are displayed. Non-significant associations include 3-year lagged divorce rate for total TAS-20 (*p* = 0.346), 3-year lagged divorce rate for DDF (*p* = 0.629), and all macrosocial indicators for EOT (all ps>0.05), which are summarized in the main text. Q_M_ = omnibus test of moderator; R^2^ = proportion of residual heterogeneity explained by the moderator.

### 3.5 Subgroup meta-regression by pandemic stages

To separate long-term secular trends from observable patterns linked to the COVID-19 pandemic, all effect sizes were assigned to three mutually exclusive time windows defined by data-collection year: pre-pandemic (2003–2018), pandemic outbreak period (2019–2022), and post-pandemic recovery phase (2023–2024). A series of mixed-effects univariate meta-regression models were fitted, with dummy-coded pandemic stage specified as the sole moderator and the pre-pandemic group serving as the reference baseline. Complete results for the three alexithymia dimensions are summarized in Table 4. The omnibus moderator test indicated that pandemic-stage grouping was associated with variation in total TAS-20, DIF, and DDF scores (all *Q_M_*, *p* < 0.001). No detectable association was observed for EOT (*Q_M_* = 2.11, *p* = 0.348, *R^2^* = 0.1%), aligning with earlier observational patterns showing that externally oriented thinking displays little covariation with macrosocial and public-health-related shifts.

**Table 4:** Adjusted mixed-effects meta-regression simultaneously including continuous collection year and post-pandemic dummy.

Outcome	*k*	Predictor	*β*	*SE*	95% CI	*p*	*Q_M_*	*R^2^*(%)
TAS-20 total	132	Collect_Year	0.17	0.09	[−0.01, 0.34]	0.061	19.89	12.1
		pandemic_dummy	3.24	1.21	[0.87, 5.6]	0.007		
DIF	77	Collect_Year	0.2	0.06	[0.08, 0.31]	<0.001	38.3	32.65
		pandemic_dummy	2.49	0.84	[0.85, 4.13]	0.003		
DDF	77	Collect_Year	0.12	0.06	[−0.003, 0.24]	0.055	83.01	51.79
		pandemic_dummy	6.35	0.91	[4.57, 8.14]	<0.001		
EOT	77	Collect_Year	0.16	0.09	[−0.02, 0.33]	0.081	3.19	1.55
		pandemic_dummy	−1.52	1.31	[−4.09, 1.05]	0.247		

(Q_M_), omnibus test for joint significance of Collect_Year + pandemic_dummy; (R^2^%), proportion of residual between-study heterogeneity explained by the two-predictor model. pandemic_dummy: 0, data collected ≤ 2022; 1, data collected 2023–2024.

For total alexithymia, pooled scores from the 2019–2022 pandemic period showed no statistically detectable difference relative to pre-pandemic levels (*β* = 0.70, *p* = 0.351). A marked score increase was observed only within the post-pandemic stage, where TAS-20 scores averaged 4.61 points higher than pre-2018 samples (*p* < 0.001). Gradual upward shifts across pandemic phases were seen for DIF and DDF. Relative to the pre-pandemic reference group, DIF scores showed moderate elevation during the pandemic period (*β* = 1.18, *p* = 0.008), with further elevation recorded for the post-pandemic period (*β* = 4.24, *p* < 0.001). DDF displayed the largest magnitude of variation across pandemic stages: a small statistically significant score increase was present for 2019–2022 (*β* = 1.00, *p* = 0.033), followed by a substantial 7.53-point elevation among post-2023 observations (*p* < 0.001). Pandemic-stage grouping accounted for more than half of residual cross-study heterogeneity for DDF (*R^2^* = 52.33%), an observational pattern showing that difficulty describing feelings exhibits the strongest covariation with pandemic-era societal conditions among the three alexithymia subdimensions. Taken together, these subgroup outputs illustrate that scores for emotion-identification and emotion-description dimensions varied across the pandemic cycle, with the highest alexithymia-related values observed in the post-pandemic recovery era. By contrast, the cognitive-avoidance dimension (EOT) remained stable across all examined time windows, showing little covariation with pandemic-linked societal disruptions.

Simple subgroup comparisons cannot fully disentangle pandemic-phase patterns from underlying secular calendar-year trends, given that all post-pandemic samples fall within the later portion of the study’s observation window. To distinguish continuous year-level secular patterns from post-pandemic-specific mean-level shifts, multivariable mixed-effects meta-regression models were fitted including both continuous data-collection year and a binary post-pandemic dummy variable (0 = ≤2022; 1 = 2023–2024). Within this model specification, the coefficient for data-collection year reflects baseline linear secular change, while the dummy-variable coefficient captures mean-level differences among post-pandemic observations after accounting for secular-year variation. Full outputs from these adjusted models are presented in Table 4. Unadjusted simple subgroup pooled estimates are provided in Supplementary Table S5 for descriptive reference only.

Several limitations apply to this three-category subgroup analysis. The calendar year 2019 represents a transitional period, preceding large-scale nationwide COVID-19-related societal changes in mainland China in early 2020. In addition, the post-pandemic group draws observations from only two calendar years (2023–2024), which may reduce the stability of raw subgroup contrasts. For these reasons, unadjusted subgroup results are retained in Supplementary Table S5 purely for descriptive purposes. Primary observational inferences regarding post-pandemic score shifts are based on the adjusted meta-regression models presented in Table 4, which incorporate continuous data-collection year as a covariate.

### 3.6 Multivariate mixed-effects meta-regression

Multivariate mixed-effects meta-regression models were fitted to examine adjusted correlational patterns among core macroeconomic indicators, after accounting for secular temporal trends and sample demographic composition. Each model simultaneously included data-collection year, female proportion of participants, 3-year lagged regional GDP (per trillion RMB), and 3-year lagged unemployment rate as covariates. Complete adjusted model outputs are summarized in Table 5. The omnibus moderator test showed that this set of predictors was jointly associated with variation in total TAS-20, DIF, and DDF scores (all *Q_M_*, *p* < 0.01). No statistically detectable association was observed for EOT (*Q_M_* = 3.72, *p* = 0.446). None of the included covariates or macroeconomic variables displayed meaningful adjusted correlations with EOT scores, which aligns with results obtained from preceding univariate and subgroup analyses.

**Table 5:** Multivariate adjusted meta-regression results.

Outcome	*k*	Predictor	*β*	*SE*	95% *CI*	*p*	*Q_M_*	*R^2^* (%)
TAS-20	128	Unemp_Lag3	1.75	0.94	[−0.09, 3.58]	0.063	13.62	7.01
DIF	75	Unemp_Lag3	1.45	0.6	[0.28, 2.62]	0.015	30.49	26.64
DDF	75	Year	−0.72	0.19	[−1.09, −0.34]	<0.001	190.24	71.73
		GDP_Lag3	0.14	0.03	[0.08, 0.2]	<0.001		
		Unemp_Lag3	4.81	0.48	[3.86, 5.75]	<0.001		

Only predictors with (*p* < 0.05) are displayed; All predictors showed no significant association with EOT (*p* > 0.05), omitted from the table. R^2^ = proportion of residual between-study heterogeneity explained by the full multivariate model.

For total alexithymia, the 3-year lagged unemployment rate presented a marginally positive adjusted correlation (*β* = 1.75, *p* = 0.063). Data-collection year, female proportion, and regional GDP showed no statistically meaningful adjusted correlations once other variables were held within the same model. For DIF (difficulty identifying feelings), unemployment rate was the only variable displaying a positive adjusted correlation (*β* = 1.45, *p* = 0.015), while the remaining covariates yielded no detectable adjusted associations. DDF (difficulty describing feelings) showed the most pronounced multivariate correlational patterns and the largest proportion of explained residual heterogeneity (*R^2^* = 71.73%). Three predictors reached statistical significance within this adjusted model: data-collection year displayed a small negative coefficient (*β* = −0.72, *p* < 0.001); regional GDP yielded a positive adjusted coefficient (*β* = 0.14, *p* < 0.001); and unemployment rate returned a large positive coefficient (*β* = 4.81, *p* < 0.001).

This negative adjusted coefficient for data-collection year regarding DDF contrasts with the positive temporal coefficient obtained from univariate meta-regression. Such coefficient-sign reversal reflects complex inter-predictor confounding, alongside potential multivariate-model instability stemming from high multicollinearity among included covariates. This counter-intuitive adjusted estimate thus requires highly cautious interpretation and is not regarded as a stable population-level observational finding within the present study.

Across different model specifications, unemployment rate consistently showed positive correlational patterns for DIF and DDF, alongside a marginally positive trend for total TAS-20. Nevertheless, this study avoids framing unemployment as a predominant correlate. This caution follows from strong inter-correlations among aggregated macrosocial indicators and the fact that many bivariate patterns are derived from independent univariate meta-regression models.

### 3.7 Sensitivity analysis and publication bias

#### 3.7.1 Publication bias

Funnel plots and Egger’s regression test were applied to assess potential publication-selection patterns for total TAS-20, DIF, DDF, and EOT. Visual examination of the four funnel plots showed an approximately symmetric distribution of effect sizes across standard-error values. Egger’s regression test for funnel-plot asymmetry returned non-significant results for total alexithymia (*z* = −0.13, *p* = 0.895), DIF (*z* = 1.34, *p* = 0.18), DDF (z = 0.51, *p* = 0.608), and EOT (z = 0.58, *p* = 0.564). Taken together, visual inspection and Egger’s regression provided no statistical indication of prominent funnel-plot asymmetry. Nevertheless, owing to the extremely high between-study heterogeneity, Egger’s test has limited capacity to identify subtle publication-selection patterns. These findings cannot definitively exclude the presence of publication bias. Complete funnel-plot graphics are available in Supplementary Figures S1–S4.

#### 3.7.2 Leave-one-out sensitivity analysis

Iterative leave-one-out exclusion was performed for the univariate temporal meta-regression model (moderator: data-collection year), to examine whether observed temporal correlational patterns depended on any single influential study.

Following each single-study removal, the year-related coefficient for total TAS-20 varied between 0.26 and 0.32, and remained statistically significant across all iterations (*p* < 0.01). For DIF, yearly slope estimates fluctuated from 0.26 to 0.30, with all iterations reaching statistical significance (*p* < 0.001). For DDF, temporal coefficients ranged from 0.29 to 0.37 and retained strong statistical significance in every iteration. For EOT, all leave-one-out models produced non-significant temporal coefficients (*p* > 0.05), with estimates spanning 0.06–0.14. No coefficient-sign reversal and no shift between significant and non-significant status occurred for any alexithymia dimension upon removal of individual effect sizes. The stable magnitude and statistical status of the temporal coefficients illustrate that the observed correlational patterns are not disproportionately shaped by any individual primary study.

#### 3.7.3 Sensitivity analysis based on studies with explicit data-collection year

A sensitivity analysis limited to studies that explicitly reported survey-implementation time was conducted to evaluate patterns when excluding cases with estimated data-collection-year values. Among the 36 eligible studies with directly reported collection time, valid total TAS-20 data were obtainable from 34 studies, and complete three-dimension data (DIF, DDF, EOT) were available for 21 studies. Temporal correlational patterns largely aligned with those derived from the full analytical sample: total TAS-20, DIF, and DDF displayed positive statistically significant temporal coefficients, whereas EOT showed no detectable temporal association. It should be noted that the dimension-specific meta-regression was based on a relatively small number of studies (*k* = 21), which may lower statistical power. Full meta-regression statistics are summarized in Supplementary Table S6.

A further sensitivity analysis excluded studies that included any postgraduate participants. The resulting temporal patterns remained highly comparable to the main analysis: positive statistically significant temporal coefficients were observed for total TAS-20, DIF, and DDF, while no meaningful temporal association emerged for EOT. Corresponding outputs are documented in Supplementary Table S3.

## 4 Discussion

The present cross-temporal meta-analysis synthesized 140 datasets covering 2003–2024 to examine secular shifts in alexithymia among Chinese college students and explore macrosocial correlates across the three TAS-20 subdimensions. Consistent with cross-sectional work highlighting concerning emotional deficits in contemporary undergraduates (Jiang et al., 2023; Zhou et al., 2022), pooled results indicated moderate overall alexithymia levels alongside a sustained long-term upward tendency. This study extends existing literature in two respects. Methodologically, the collection-year-anchored cross-temporal design captures two decades of emotional dynamics, mitigating static limitations of single-time-point surveys. In sampling terms, this nationwide multi-year dataset reduces sampling bias common in small localized investigations and yields more generalizable population-level clues for youth alexithymia research.

Meta-regression results revealed dimensional differentiation in secular alexithymia trends: difficulties identifying feelings and difficulties describing feelings rose across the study period, whereas externally oriented thinking remained relatively stable. Leave-one-out sensitivity tests suggested these temporal patterns were not dependent on a small subset of outlier studies, pointing toward potential cohort-level differences in emotional-processing capacities rather than random sampling fluctuation. Beyond continuous yearly trends, pandemic-related subgroup analyses yielded tentative observational patterns across pre-pandemic, pandemic-era, and post-pandemic periods. Pandemic-stage grouping is inherently confounded with calendar time and parallel societal shifts; accordingly, post-2023 score elevations cannot be definitively attributed to public-health-related societal disruptions. Restricted offline social interaction, lingering epidemic-related anxiety, and disrupted in-person family communication serve as plausible post-hoc hypotheses for contextualizing these aggregate-level observations, though these putative mechanisms were not directly tested in included primary studies. External-thinking-related cognitive tendencies showed minimal variation across pandemic phases, tentatively suggesting societal disturbances may primarily correlate more strongly with affective processing rather than deep-seated cognitive dispositions.

Univariate lagged meta-regression detected positive observational correlations between several macrosocial indicators and elevated alexithymia-related scores. These represent purely correlational patterns. Given risks of ecological inference and temporal confounding among inter-correlated societal metrics, causal inferences cannot be drawn; these exploratory associations reflect co-occurring secular population-level patterns that can be contextualized through social-ecological and social-learning frameworks.

Mechanistic accounts below constitute plausible post-hoc hypotheses for interpreting aggregate-level temporal covariation and were not empirically validated in the included literature. Socio-economic transformations such as rising housing, education, and medical costs may co-occur with shifts in youth emotional-cognitive profiles. Household financial strain may coincide with more frequent inter-parental conflict and negative domestic atmospheres. Adolescents growing within such environments may receive limited emotional support and adopt maladaptive emotion-regulation strategies, which may correlate with poorer interpersonal functioning and higher alexithymia-related traits (Peng et al., 2018). Heightened academic competition and graduate-employment pressure may also narrow opportunities for young adults to practice emotional expression, correlating with greater emotion-processing deficits (Su and Liu, 2020).

Shifting family structures represent another plausible source of youth emotional vulnerability. Higher divorce rates may increase the share of single-parent and multigenerational households, potentially eroding stable parental companionship and core family-bonding functions (Wu et al., 2018). The family ecosystem framework regards intact family structure and secure emotional attachment as important protective factors for adolescent mental development (Li and Zou, 2006). Large-scale social transformation may gradually weaken such protective emotional buffers, leaving young people more susceptible to alexithymia-related traits.

From an information-ecology perspective, broad internet penetration may also correlate with emotional dysfunction in college-student samples. Unfiltered mass negative online content can distort interpersonal cognition and foster mild chronic social mistrust (Hawkley and Cacioppo, 2010). Although digital platforms expand superficial social contact, loose homogeneous virtual ties often fail to satisfy deep emotional-attachment needs (Zhai, 2018). Prolonged virtual-environment engagement reduces offline emotion-communication practice, and digital-addictive tendencies may further amplify deficits in identifying and describing feelings (Cheng et al., 2023). Taken together, informatization may display population-level temporal covariation alongside rising alexithymia-related scores among young cohorts, representing one among several plausible long-term structural correlates.

Multivariate adjusted meta-regressions produced differentiated conditional correlational patterns after controlling for data-collection year and sample gender composition. Severe multicollinearity across national-level societal metrics means multivariate coefficients cannot be treated as fully disentangled independent effects. Within this specific model specification, lagged GDP and unemployment showed conditional correlational patterns with emotion-description deficits, while unemployment exhibited a conditional correlational pattern for difficulty identifying feelings. No tested socioeconomic indicator showed meaningful associations with externally oriented thinking, reiterating that this cognitive sub-dimension appears less responsive to macrosocial stressors. The relatively weak link between urban unemployment rates and overall alexithymia may partly stem from shifting graduate-employment attitudes and mismatches in official statistical calibers (Tang, 2024), which can weaken observable connections between macro employment metrics and college-student emotional status.

Proportion of female participants in included samples showed no consistent independent predictive association with any alexithymia dimension after covariate adjustment. This aligns with existing meta-analyses on Chinese adolescents and young adults (Wang et al., 2024; Zhong et al., 2026), which reported negligible pooled gender gaps despite small raw prevalence differences. Most primary publications only supplied aggregated total alexithymia scores without gender-stratified subscale outputs, restricting exploration of fine-grained subgroup differences and introducing potential unmeasured bias. Synthesized evidence cannot establish conclusive stable gender differences in alexithymia for Chinese college students; further high-quality primary studies reporting disaggregated gender-specific subscale data are needed to resolve this question.

Two complementary sets of robustness checks were implemented to evaluate the reliability of core observational findings. Egger’s regression asymmetry tests yielded little indication of severe publication bias across the four alexithymia dimensions, and complete funnel plots are provided in Supplementary materials. Sequential leave-one-out sensitivity analyses demonstrated that the magnitude and statistical significance of temporal trends remained largely stable after removing each individual study. Temporal slope estimates for alexithymia-related dimensions proved robust across multiple sensitivity analyses. Even so, considering extreme between-study heterogeneity (*I^2^* > 99.5%), temporal-coding uncertainty, and possible measurement-practice variation across investigations, absolute pooled mean alexithymia values show substantial dispersion and cannot be regarded as broadly stable. Core observed patterns are unlikely to be depend exclusively by reporting bias or single influential outlier studies.

## 5 Limitations and future directions

This meta-analysis has several important inherent limitations that suggest valuable avenues for future research.

All included primary studies exclusively used the TAS-20 to assess alexithymia, leaving other well-validated instruments underexplored, including the Observer Alexithymia Scale (OAS; Haviland and Reise, 1996), the Perth Alexithymia Questionnaire (PAQ; Preece et al., 2020), and the Bermond-Vorst Alexithymia Questionnaire (BVAQ; Vorst and Bermond, 2001). Future cross-temporal meta-analyses incorporating data from these alternative scales could enable cross-instrument comparisons to test whether the secular temporal patterns observed in this study hold across different measurement tools.

Generalizability of findings is constrained by the composition of available study samples. All pooled effect sizes are based on full-time undergraduate and junior-college students; accordingly, the observed temporal patterns cannot be readily generalized to other Chinese young-adult subgroups such as vocational trainees, non-student youth, and migrant young workers.

Approximating data-collection year by subtracting 2 years from publication year may introduce temporal misclassification and measurement error. This concern was addressed through a sensitivity analysis restricted to the 36 studies with explicitly reported survey-collection dates. Although main temporal trends were replicated within this subset, statistical power for sub-dimension analyses was limited (*k* = 21). These sensitivity outputs are reported alongside primary findings, which calls for cautious interpretation of secular trends derived from the full dataset.

This meta-analysis demonstrated extreme between-study heterogeneity (*I^2^* > 99.5%) across all alexithymia outcomes. Even after accounting for data-collection year as a temporal moderator, substantial residual heterogeneity remained unexplained. Potentially meaningful moderators such as regional context, university sampling characteristics, and subtle procedural differences in Chinese TAS-20 implementation could not be systematically extracted from original publications. Wide prediction intervals further indicated large dispersion of true effect sizes across primary studies. For this reason, we avoid over-interpreting absolute pooled mean alexithymia values; our core conclusions focus on temporal cohort-level patterns rather than absolute-level estimates.

National-level macrosocial meta-regression results are subject to multiple methodological caveats. Aggregate national-level metrics carry risks of the ecological-inference fallacy: associations observed at the population level may not translate to individual-student-level relationships. Most socioeconomic indicators rise monotonically across calendar time and exhibit high inter-correlation; applying 3-year or 5-year lags cannot fully resolve temporal confounding and cannot establish causal or temporally ordered effects. The large number of predictor-outcome combinations inflates Type-I error risk, so all macrosocial outputs should be regarded as purely exploratory observational correlations, not confirmatory causal evidence. High multicollinearity among aggregated macrosocial indicators also prevents fitting one fully adjusted multivariate meta-regression containing all predictors simultaneously. When multiple effect sizes originate from a single primary study, potential non-independence of observations cannot be fully addressed; robust variance estimation could not be reliably implemented given the dataset structure. Adjusted meta-regression separating secular trends and post-pandemic shifts also carries constraints, as post-pandemic observations are limited to 2023–2024 and produce partial collinearity between continuous collection year and the post-pandemic dummy variable. Findings related to post-pandemic-specific shifts therefore remain exploratory rather than definitive.

Assessments of publication bias in the present work have notable limitations. Our literature search omitted gray-literature sources and did not perform forward- or backward-citation chasing, meaning unpublished null or small-effect studies may be missing from the dataset. Under extreme between-study heterogeneity, Egger’s regression test has low sensitivity for detecting subtle funnel-plot asymmetry, so subtle publication-selection bias cannot be fully ruled out.

Conclusions from this meta-analysis are restricted to Chinese youth cohorts. To evaluate whether rising alexithymia and its macrosocial correlates generalize cross-culturally, future cross-temporal meta-analyses sampling adolescent and college-student groups from diverse cultural backgrounds would provide informative comparative evidence.

## 6 Conclusion

Drawing on 140 eligible studies with data collected between 2003 and 2024, this cross-temporal meta-analysis observed moderate average alexithymia levels among Chinese college students. The pooled data indicated gradual upward tendencies in total TAS scores, DIF and DDF across the sampling window, with a potential additional rise in emotional deficits recorded during the post-2023 pandemic recovery phase, while EOT scores showed little measurable temporal fluctuation. Lagged GDP per capita, housing prices, divorce rates and internet penetration displayed positive correlations with elevated alexithymic symptoms. After controlling for relevant covariates, lagged GDP and unemployment showed unique statistical associations only with DDF, and unemployment showed an independent statistical association with DIF. None of the examined macrosocial indicators exhibited meaningful statistical correlations with EOT, and the proportion of female participants within samples showed no consistent predictive pattern for any alexithymia dimension. Follow-up sensitivity analyses and Egger’s regression tests suggested the full set of analytic results were relatively stable and unlikely to be meaningfully biased by publication bias or single influential individual studies. Collectively, the correlational patterns implied that broad socioeconomic transformations and pandemic-related societal changes may disproportionately correlate with young adults’ difficulties in identifying and articulating internal emotions, rather than showing covariation with shifts in externally-oriented cognitive styles. The observational patterns reported here may provide population-scale empirical reference for designing targeted emotional support programs within university mental health services.

## Data Availability

The full study‑level dataset supporting this meta‑analysis is available in Supplementary Table S2 within the Supplementary Material. R analytical scripts for temporal modelling, multivariate meta‑regression, sensitivity analyses, and publication‑bias diagnostics have been deposited on the Open Science Framework (OSF). The OSF repository also hosts an archival backup copy of the extracted dataset (Data Extraction Table of Included Studies.xlsx). The archived OSF project resource includes complete descriptive information for all included studies, standardized meta‑analytic input data, and reproducible code for all temporal, multivariate, sensitivity, and publication‑bias analyses. The publicly accessible project page can be found at: https://osf.io/4d5pt/. Further inquiries can be directed to the corresponding author.

## References

[ref1] Bronfenbrenner U. (1979). The Ecology of human Development: Experiments by Nature and design. Cambridge, MA: Harvard University Press.

[ref2] Cai H. Huang Z. Lin L. Jing Y. (2020). The psychological change of the Chinese people over the past half century: a literature review. Adv. Psychol. Sci. 28, 1599–1618. doi: 10.3724/SP.J.1042.2020.01599 [In Chinese]

[ref3] Chen B. Sun X. (2022). Cross-temporal changes of college students’ time management disposition in the mainland of China during 1999~2020. Adv. Psychol. Sci. 30, 1968–1988. doi: 10.3724/SP.J.1042.2022.01968 [In Chinese]

[ref4] Cheng P. Zhang H. Ge W. Liu Y. (2023). The effect of alexithymia on mobile phone addiction: the sequential mediating effect of negative evaluation fear and self-control. Chin. J. Clin. Psychol. 31, 593–597. [In Chinese]

[ref6] Edwards D. J. Lowe R. (2021). Associations between mental health, interoception, psychological flexibility, and self-as-context, as predictors for alexithymia: a deep artificial neural network approach. Front. Psychol. 12:637802. doi: 10.3389/fpsyg.2021.637802, 33868110 PMC8044902

[ref7] Fang Y. Mai Y. Wu X. Zhao J. (2020). The relationship between alexithymia and suicide risk in undergraduates: the chain mediating effect of impulsivity and aggressivity. Chin. J. Spec. Educ. 2, 63–68. [In Chinese]

[ref8] Haviland M. G. Reise S. P. (1996). A California Q-set alexithymia prototype and its relationship to ego-control and ego-resiliency. J. Psychosom. Res. 41, 597–607. 10.1016/s0022-3999(96)00223-1 9032723

[ref9] Hawkley L. C. Cacioppo J. T. (2010). Loneliness matters: a theoretical and empirical review of consequences and mechanisms. Ann. Behav. Med. 40, 218–227. doi: 10.1007/s12160-010-9210-8, 20652462 PMC3874845

[ref10] Hemming L. Shaw J. Haddock G. Carter L.-A. Pratt D. (2021). A cross-sectional study investigating the relationship between alexithymia and suicide, violence, and dual harm in male prisoners. Front. Psychol. 12:670863. doi: 10.3389/fpsyt.2021.670863, 33995152 PMC8116549

[ref11] Hou Y. Zhang S. Hou Y. Xu C. (2021). Role of interaction anxiousness and alexithymia between mobile phone addiction and life satisfaction of freshmen. Occup. Health 37, 88–91. [In Chinese]

[ref12] Jiang H. Liang H. Li B. Tuo A. (2023). Alexithymia and mobile phone addiction among college students: mediation by boredom proneness and anxiety. J. Psychol. Afr. 33, 132–137. doi: 10.1080/14330237.2023.2190223

[ref13] Ledermann K. von Känel R. Barth J. Schnyder U. Znoj H. Schmid J. P. . (2020). Myocardial infarction-induced acute stress and post-traumatic stress symptoms: the moderating role of an alexithymia trait–difficulties identifying feelings. Eur. J. Psychotraumatol. 11:1804119. doi: 10.1080/20008198.2020.1804119, 33488995 PMC7803082

[ref14] Li S. Chen X. Liu L. Sun C. (2024). The relationship between social withdrawal and problematic social media use in Chinese college students: a chain mediation of alexithymia and negative body image. BMC Psychol. 12:246. doi: 10.1186/s40359-024-01755-0, 38702805 PMC11067283

[ref15] Li C. Zou H. (2006). Relationship between the teenager’s sense of isolation and the function of personality and family. J. Shanxi Normal University (Philos. Soc. Sci. Edition) 35, 115–121. [In Chinese]

[ref16] Liu H. Y. Wang W. (2019). Relationship between autism traits and suicide idea: the mediating role of alexithymia and depression. Chin. J. Clin. Psychol. 27, 889–893. [In Chinese]

[ref17] Lumley M. A. Neely L. C. Burger A. J. (2007). The assessment of alexithymia in medical settings: implications for understanding and treating health problems. J. Pers. Assess. 89, 230–246. doi: 10.1080/00223890701629698, 18001224 PMC2931418

[ref18] Mendia J. Zumeta L. N. Cusi O. Pascual A. Alonso-Arbiol I. Díaz V. . (2024). Gender differences in alexithymia: insights from an updated meta-analysis. Pers. Individ. Differ. 227:112710. doi: 10.1016/j.paid.2024.112710

[ref19] Morie K. P. Yip S. W. Nich C. Hunkele K. Carroll K. M. Potenza M. N. (2016). Alexithymia and addiction: a review and preliminary data suggesting neurobiological links to reward/loss processing. Curr. Addict. Rep. 3, 239–248. doi: 10.1007/s40429-016-0097-8, 27695665 PMC5040368

[ref20] Page M. J. McKenzie J. E. Bossuyt P. M. Boutron I. Hoffmann T. C. Mulrow C. D. . (2021). The PRISMA 2020 statement: an updated guideline for reporting systematic reviews. BMJ 372:n71. doi: 10.1136/bmj.n71, 33782057 PMC8005924

[ref21] Pearlin L. I. Menaghan E. G. Lieberman M. A. Mullan J. T. (1981). The stress process. J. Health Soc. Behav. 22, 337–356. 7320473

[ref22] Peng Y. Zhu L. Wang Z. (2018). Association between parental emotion expressiveness and adolescents’ behavior problem: the mediating role of the parent-child attachment and loneliness. Psychol. Dev. Educ. 34, 504–512. [In Chinese]

[ref23] Preece D. A. Becerra R. Allan A. Robinson K. Chen W. Hasking P. . (2020). Assessing alexithymia: psychometric properties of the Perth alexithymia questionnaire and 20-item Toronto alexithymia scale in United States adults. Pers. Individ. Differ. 166:110138. doi: 10.1016/j.paid.2020.110138

[ref24] Prins J. Blanker M. H. Bohnen A. M. Thomas S. Bosch J. L. H. R. (2002). Prevalence of erectile dysfunction: a systematic review of population-based studies. Int. J. Impot. Res. 14, 422–432. doi: 10.1038/sj.ijir.3900905, 12494273

[ref25] Shi L. Jiao J. Mu D. (2023). A cross-temporal meta-analysis of changes of resilience level in Chinese college students. Chin. Ment. Health J. 37, 894–898. [In Chinese]

[ref26] Sifneos P. E. (1996). Alexithymia: past and present. Am. J. Psychiatry 153, 137–142. 10.1176/ajp.153.7.137 8659637

[ref27] Su Q. Liu G. (2020). Depression in Chinese adolescents from 1989 to 2018: an increasing trend and its relationship with social environments. Curr. Psychol. 41, 6966–6977. doi: 10.1007/s12144-020-01181-6, 30311153

[ref28] Tang S. (2024). Employment attributes, current status, and characteristics of China’s youth population. People's Tribune 12, 32–34. [In Chinese]

[ref29] Taylor G. J. (2000). Recent developments in alexithymia theory and research. Can. J. Psychiatr. 45, 134–142. 10.1177/070674370004500203 10742872

[ref30] Twenge J. M. (2000). The age of anxiety? The birth cohort change in anxiety and neuroticism, 1952–1993. J. Pers. Soc. Psychol. 79, 1007–1021. 10.1037//0022-3514.79.6.1007 11138751

[ref31] Vorst H. C. Bermond B. (2001). Validity and reliability of the Bermond–Vorst alexithymia questionnaire. Pers. Individ. Differ. 30, 413–434. doi: 10.1016/S0191-8869(00)00033-7

[ref32] Wang X. Li X. Guo C. Hu Y. Xia L. Geng F. . (2021). Prevalence and correlates of alexithymia and its relationship with life events in Chinese adolescents with depression during the COVID-19 pandemic. Front. Psychol. 12:774952. doi: 10.3389/fpsyt.2021.774952, 34880795 PMC8645693

[ref33] Wang G. Wuyun T. N. Jin T. L. (2024). A cross-temporal meta-analysis of alexithymia among Chinese adolescents. J. Inner Mongolia Normal University (Natural Sci. Edition) 53, 78–84. [In Chinese]

[ref34] Wu Y. Wang P. Du S. (2018). China’s changing family structure and adolescent development. Soc. Sci. China 2, 98–120. [In Chinese]

[ref35] Xin S. Wang Y. (2019). A cross-temporal meta-analysis of changes in Chinese college students’ achievement motivation during 1999-2014. Psychol. Dev. Educ. 35, 288–294. [In Chinese]

[ref36] Xin Z. Xin S. Zhang M. (2011). Changes in college students' anxiety level in 1993-2009: a cross-temporal meta-analysis. Psychol. Dev. Educ. 27, 648–653. [In Chinese]

[ref37] Yi J. Yao S. Zhu X. (2003). The Chinese version of the TAS-20: reliability and validity. Chin. Ment. Health J. 17, 763–767. [In Chinese]

[ref38] Yuan Y. Shen X. Zhang X. Li H. (2003). The reliability and validity of Toronto alexithymia scale (TAS-20). Sichuan Mental Health 16, 25–27. [In Chinese]

[ref39] Zhai G. (2018). Spatiotemporal transformation of network society and risk aversion of teenagers socialization. J. Anhui Normal University (Human. Soc. Sci.) 47, 111–116. [In Chinese]

[ref40] Zhang Y. L. Li S. Yu G. L. (2020). Alexithymia and aggression among adolescents: the mediating role of boredom proneness. Chin. J. Clin. Psychol. 28, 383–386. [In Chinese]

[ref41] Zhang C. Zhang J. Zhang J. Zhang P. (2011). A review of researches on cognitive and social mechanism of alexithymia. Adv. Psychol. Sci. 19, 398–409. [In Chinese]

[ref42] Zhong Y. Chen S. Zeng Q. Ku M. Zhang Y. Lyu J. (2026). Prevalence of alexithymia in Chinese adolescents: a systematic review and meta-analysis. BMC Psychiatry 26:38. doi: 10.1186/s12888-025-07651-0, 41530720 PMC12801485

[ref43] Zhou Y. Dai L. Deng Y. Zeng H. Yang L. (2022). The moderating effect of alexithymia on the relationship between stress and cyberchondria. Front. Psychol. 13:1043521. doi: 10.3389/fpsyt.2022.1043521, 36386983 PMC9640749

