# Supplementary material for: Secular temporal shifts in alexithymia among Chinese college students (2003–2024): a cross-temporal meta-analysis
Source: Front Psychol. 2026 Sep 25;17:1945943. doi: 10.3389/fpsyg.2026.1945943 (PMC13649997; doi:10.3389/fpsyg.2026.1945943)
Supplement: Supplementary file 1 [file Table_3.DOCX]

Table S3 Sensitivity analysis: meta‑regression of alexithymia dimensions on data‑collection year, excluding studies with postgraduate participants

| Outcome | *k* | *β* (year) | *SE* | 95% CI | *Q_M_* | *p* (Q_M_) | *R²* (%) |
| --- | --- | --- | --- | --- | --- | --- | --- |
| TAS‑20 total | 129 | 0.31 | 0.08 | [0.15, 0.47] | 14.08 | <0.001 | 9.28 |
| DIF | 74 | 0.29 | 0.06 | [0.17, 0.40] | 24.62 | <0.001 | 24.64 |
| DDF | 74 | 0.37 | 0.08 | [0.21, 0.52] | 22.1 | <0.001 | 22.56 |
| EOT | 74 | 0.09 | 0.09 | [-0.08, 0.27] | 1.06 | 0.303 | 0.08 |

Notes. *β* (year) = unstandardized regression coefficient for data‑collection year; SE = standard error; CI = 95% confidence interval; *Q_M_* = omnibus moderator test statistic; *R²* = percentage of residual between‑study heterogeneity explained by data‑collection year.
