# Supplementary material for: Secular temporal shifts in alexithymia among Chinese college students (2003–2024): a cross-temporal meta-analysis
Source: Front Psychol. 2026 Sep 25;17:1945943. doi: 10.3389/fpsyg.2026.1945943 (PMC13649997; doi:10.3389/fpsyg.2026.1945943)
Supplement: Supplementary file 2 [file Table_4.DOCX]

| Outcome | Pooled Mean | 95% *CI‑lb* | 95% *CI‑ub* | 95% *PI‑lb* | 95% *PI‑ub* | *τ²* | *I^2^* (%) |
| --- | --- | --- | --- | --- | --- | --- | --- |
| TAS‑20 total | 54.29 | 53.57 | 55.02 | 45.98 | 62.61 | 17.86 | 99.58 |
| DIF | 18.9 | 18.43 | 19.37 | 14.74 | 23.06 | 4.44 | 99.79 |
| DDF | 14.99 | 14.39 | 15.6 | 9.63 | 20.36 | 7.39 | 99.88 |
| EOT | 21.73 | 21.12 | 22.35 | 16.33 | 27.14 | 7.50 | 99.93 |

Table S4. Pooled Random‑Effects Model Estimates for Alexithymia Outcomes

Note. CI = 95% confidence interval for the overall pooled mean score; *PI* = 95% prediction interval, indicating the expected range of true effect sizes for future independent primary studies; *τ²* = between‑study variance; *I^2^*  = proportion of total variance attributable to between‑study heterogeneity.
