# Supplementary material for: Secular temporal shifts in alexithymia among Chinese college students (2003–2024): a cross-temporal meta-analysis
Source: Front Psychol. 2026 Sep 25;17:1945943. doi: 10.3389/fpsyg.2026.1945943 (PMC13649997; doi:10.3389/fpsyg.2026.1945943)
Supplement: Supplementary file 3 [file Table_5.DOCX]

Supplementary Table S5 Subgroup Meta-Regression by Pandemic Stages

| Outcome | *k* | *Moderator* | *β* | *SE* | 95% *CI* | *p* | *Q_M_* | *R^2^* (%) |
| --- | --- | --- | --- | --- | --- | --- | --- | --- |
| TAS-20 | 132 | Post-pandemic vs Pre-pandemic | 4.61 | 1.12 | [2.41, 6.82] | <0.001 | 16.94 | 10.34 |
| DIF | 77 | Pandemic vs Pre-pandemic | 1.18 | 0.44 | [0.31, 2.05] | 0.008 | 32.34 | 28.82 |
|  |  | Post-pandemic vs Pre-pandemic | 4.24 | 0.78 | [2.72, 5.77] | <0.001 |  |  |
| DDF | 77 | Pandemic vs Pre-pandemic | 1 | 0.47 | [0.08, 1.92] | 0.033 | 84.81 | 52.33 |
|  |  | Post-pandemic vs Pre-pandemic | 7.53 | 0.82 | [5.92, 9.13] | <0.001 |  |  |

Notes. Reference group = 2003–2018 pre-pandemic period. Pandemic = 2019–2022; Post-pandemic = 2023–2024. Only statistically significant results (*p* < 0.05) are presented. Non-significant comparisons and full results for the EOT subscale are summarized in the main text. *β* = unstandardized mean difference relative to the pre-pandemic baseline. *Q_M_* = joint moderator test statistic; *R^2^* = proportion of residual between-study heterogeneity explained by pandemic staging.
