# Supplementary material for: Secular temporal shifts in alexithymia among Chinese college students (2003–2024): a cross-temporal meta-analysis
Source: Front Psychol. 2026 Sep 25;17:1945943. doi: 10.3389/fpsyg.2026.1945943 (PMC13649997; doi:10.3389/fpsyg.2026.1945943)
Supplement: Supplementary file 4 [file Table_6.DOCX]

| Outcome | *k* | *β* (year) | *SE* | 95% CI | *Q_M_* | *p* (*Q_M_*) | *R²* (%) |
| --- | --- | --- | --- | --- | --- | --- | --- |
| TAS‑20 total | 34 | 0.70 | 0.19 | [0.35, 1.05] | 15.57 | <0.001 | 30.7 |
| DIF | 21 | 0.47 | 0.14 | [0.21, 0.74] | 12.23 | <0.001 | 36.06 |
| DDF | 21 | 0.78 | 0.17 | [0.45, 1.11] | 21.43 | <0.001 | 50.57 |
| EOT | 21 | 0.30 | 0.22 | [-0.13,0.74] | 1.87 | 0.171 | 4.21 |

Supplementary Table S6 Sensitivity analysis: temporal meta‑regression restricted to studies with explicitly reported data‑collection year

Note. Only studies providing explicit data‑collection time were included in this sensitivity analysis. *k* = number of effect sizes. *β* (year): regression coefficient for data‑collection year; *Q_M_*: omnibus test of moderators; *R²*: proportion of heterogeneity explained by data‑collection year.
